# Unique Combination of Diamond–Blackfan Anemia and Lynch Syndrome in Adult Female: A Case Report

**DOI:** 10.3389/fonc.2021.652696

**Published:** 2021-04-16

**Authors:** Aleksey S. Tsukanov, Dmitriy Y. Pikunov, Vitaly P. Shubin, Aleksey A. Barinov, Vladimir N. Kashnikov, Yuri A. Shelygin, Andrey D. Kaprin, Elena V. Filonenko, Dmitriy V. Sidorov, Aleksey A. Maschan, Galina A. Novichkova, Liudmila A. Yasko, Elena V. Raykina, Aleksandr G. Rumyantsev

**Affiliations:** ^1^ Ryzhikh National Medical Research Center of Coloproctology, Moscow, Russia; ^2^ National Medical Radiology Research Center, Moscow, Russia; ^3^ Dmitry Rogachev National Medical Research Center of Pediatric Hematology, Oncology and Immunology, Moscow, Russia

**Keywords:** endometrial cancer, colorectal cancer, Diamond–Blackfan anemia, Lynch syndrome, microsatellite instability, NGS, aCGH

## Abstract

We present an extremely rare clinical case of a 38-year-old Russian patient with multiple malignant neoplasms of the uterus and colon caused by genetically confirmed two hereditary diseases: Diamond–Blackfan anemia and Lynch syndrome. Molecular genetic research carried out by various methods (NGS, Sanger sequencing, aCGH, and MLPA) revealed a pathogenic nonsense variant in the *MSH6* gene: NM_000179.2: c.742C>T, p.(Arg248Ter), as well as a new deletion of the chromosome 15’s locus with the capture of 82,662,932–84,816,747 bp interval, including the complete sequence of the *RPS17* gene. The lack of expediency of studying microsatellite instability in endometrial tumors using standard mononucleotide markers NR21, NR24, NR27, BAT25, BAT26 was demonstrated. The estimated prevalence of patients with combination of Diamond–Blackfan anemia and Lynch syndrome in the world is one per 480 million people.

## Introduction

Diamond–Blackfan anemia (DBA) is a rare genetically determined form of congenital bone marrow failure syndromes characterized by a block of predominant erythropoiesis, the presence of multiple congenital anomalies, malformations, and a predisposition for the development of tumors (1). According to the Russian registry, the annual incidence of DBA is 6.3 ± 0.3 cases per 1,000,000 live births (2). In more than 70% of cases patients with a clinical diagnosis of DBA have a defect in the genes encoding proteins of the small and large ribosomal subunits, which leads to an imbalance in the synthesis of ribosomal RNA and ribosomal proteins and, as a result, to the activation and stabilization of the p53 protein, disruption of the cell cycle and apoptosis (3, 4). Most often, heterozygous mutations are detected in the following ribosomal protein genes: *RPS19*, *RPS10*, *RPS24*, *RPS26*, *RPL5*, *RPL11*, *RPL35a*, *RPS7*, *RPS17*. In addition, there are described cases caused by biallelic mutations in the *ADA2*, *EPO*, *FLVCR1* genes, mutations in the genes located on the X chromosome: *TSR2*, *GATA1* (5–7). The cumulative risk of developing malignant neoplasms in patients with DBA exceeds the general population risk by 5.4 times. The maximum risk was noted for myelodysplastic syndrome, acute myeloid leukemia, colon adenocarcinoma, osteosarcoma, and malignant tumors of the female genital organs (8).

Lynch syndrome (LS), caused by germline mutations in genes of the DNA mismatch repair system (MMR), is the most common hereditary cancer syndrome. The population frequency of this syndrome in Europeans is 1:500–1:1,000 (9). Basically, mutation carriers develop colon and endometrial cancers, but tumors can also occur in other organs: ovaries, brain, sebaceous glands, stomach, *etc.* (10). Heterozygous germline mutations are most often found in the *MLH1* and *MSH2* genes, less often in the *MSH6* and *PMS2* genes (11). Genetic variants leading to biallelic inactivation of the listed genes cause the development of a rare autosomal recessive syndrome—Constitutional Mismatch Repair Deficiency syndrome (CMMRD). In most patients with CMMRD multiple primary tumors (gliomas, hematologic malignancies, colorectal cancer) develop during childhood (12). Tumors of patients with MMR defect are microsatellite unstable. This phenomenon occurs only in ~15% of all colorectal cancer cases, which makes it possible to screen out patients with microsatellite stable tumors already at the first stage of the diagnosis of Lynch syndrome (11, 13). Various criteria are used to select patients with suspected Lynch syndrome, taking into account the clinical characteristics of the patients, of which Amsterdam II and Bethesda are the best known (10). The Russian Federation also proposed its own selection criteria: the occurrence of colorectal cancer in patients under the age of 43; along with colorectal cancer two (or more) cases of cancer of any localization in the patient or his relatives (14). Tactics of diagnostics, surgical treatment, and clinical monitoring have been developed for patients with LS. This makes it possible to prevent the development of advanced forms of the disease, increasing the life span of patients (10).

Here, we report the unique case of a patient with a combination of two hereditary diseases: Diamond–Blackfan anemia and Lynch syndrome, either of which, individually, can cause the development of tumors of an epithelial and hematopoietic nature. After advanced search in available literature, we did not find any information about such rare combination of these hereditary cancer syndromes.

## Material and Methods

### Case History

A 38-year-old female patient (A.) was admitted to the National Medical Radiology Research Center of the Ministry of Health of the Russian Federation in March 2020 with complaints of alternating constipation and diarrhea, recurrent bloating, blood and mucus in stool. The patient signed informed agreement to undergo diagnostic procedures and treatment, as well as to participate in the study, and for the presentation of clinical and molecular data in scientific and medical literature. This case report was approved by the local Ethics Committee at the National Medical Radiology Research Center, Moscow, Russia.

**Figure 1:**
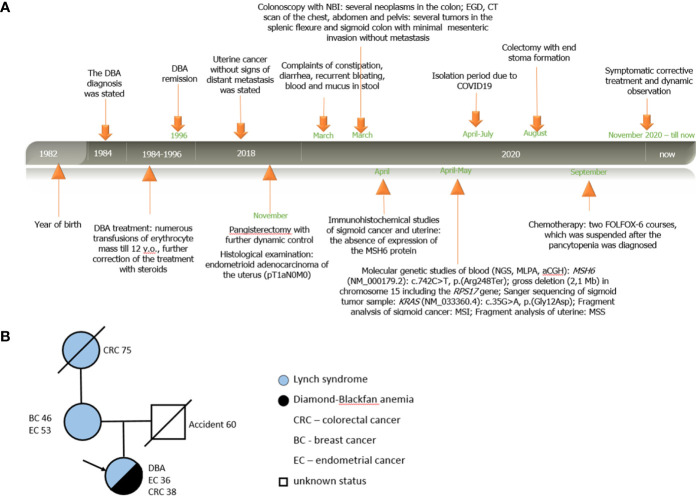
Personal and family history of patient A. **(A)** Timeline. Relevant data from diagnosis statement till now organized as a timeline; **(B)** Pedigree structure of affected family.

According to the life history, the patient since her childhood was under dynamic supervision of a hematologist at Dmitry Rogachev National Medical Research Center of Pediatric Hematology, Oncology and Immunology (Moscow, Russia) for DBA, which has debuted with normochromic normocytic anemia in the first year of life without signs of hemolysis and micronutrient deficiency. The diagnosis was confirmed by the bone marrow puncture, which revealed a reduction of the erythroid lineage with the preservation of the myeloid and megakaryocytic lineages of hematopoiesis. Physical examination did not reveal any congenital anomalies or malformation characteristic of DBA. As a symptomatic treatment, the patient underwent numerous transfusions of erythrocyte mass till 12 y.o.; further correction of the treatment with steroids made it possible to achieve transfusion independence. In 1996 (at the age of 14), stable remission of the disease without clinical manifestations was stated. At the age of 36 (in 2018), the patient was diagnosed with uterine cancer without signs of distant metastasis and underwent pangisterectomy at the oncological unit at her place of residence. Histological examination revealed the presence of endometrioid adenocarcinoma of the uterus G1 in stage IA (pT1aN0M0). No adjuvant treatment was performed; the patient was under dynamic control (**Figure 1A**).

According to the clinical family history analysis, the patient’s grandmother (from the mother’s side) was diagnosed with sigmoid cancer at the age of 75; the patient’s mother had a breast cancer at the age of 46, then at the age of 53 she had uterine cancer (treated surgically), and now she is alive without signs of disease progression. The patient’s father died in an accident at the age of 60 with no signs of previous hereditary disease or cancer. The patient has no other relatives with oncological history (**Figure 1B**).

### Instrumental Diagnosis

**Figure 2:**
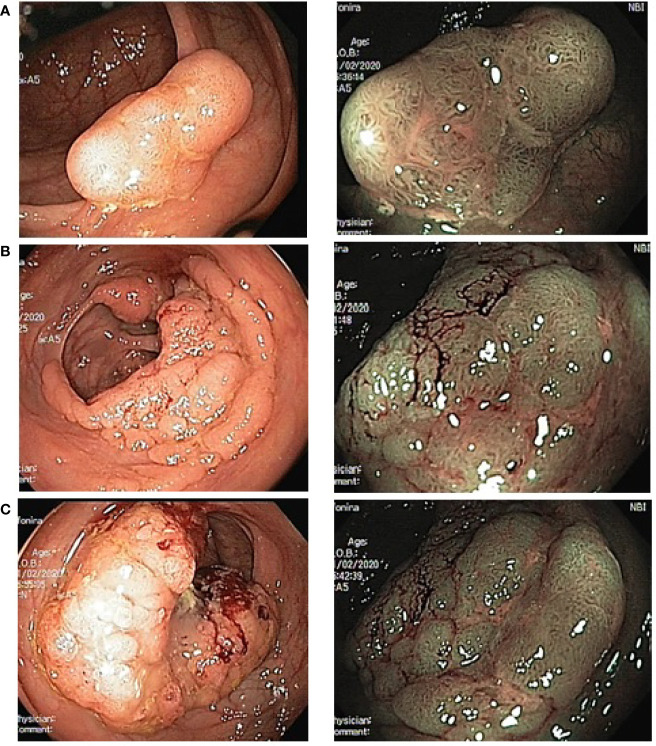
Endoscopic image of multiple colonic tumors in the patient A. (left photo—standard colonoscopy, right photo—colonoscopy with NBI). **(A)**—adenoma in cecum; **(B)**—malignant tumor in splenic flexure; **(C)**—malignant tumor in sigmoid.

Patient A. was examined at the Medical Radiology Research Center. Colonoscopy with narrow band imaging (NBI) detected several neoplasms in the colon: broad-base adenoma with a diameter of 13 mm in the cecum (type 2 according to the NICE classification); two flat adenomas 8 × 5 mm and 6 × 6 mm in the ascending colon (also type 2 according to the NICE classification); an epithelial formation occupying 3/4 of the intestinal circumference and extending for two haustras in the splenic flexure; a large rigid tumor with ulceration, pathological vessels, and irregular architectonics, occupying 3/4 of the bowel circumference in the sigmoid colon at the level of ~35 cm from the anus; a flat epithelial mass with a diameter of 23 mm in the distal sigmoid colon (**Figure 2**).

Comprehensive follow-up examination (EGD, CT scan of the chest, abdomen, and pelvis) confirmed the presence of tumors in the splenic flexure and sigmoid colon with minimal (2–3 mm) mesenteric invasion without signs of regional and distant metastasis. No additional diagnostic findings have been identified.

Taking into account anamnestic data with previous uterine cancer at the age of 36 y.o., the presence of synchronous colonic tumors, clinical family history data, after genetic counseling the patient underwent complex immunohistochemical and molecular genetic studies at Ryzhikh National Medical Research Center of Coloproctology (Moscow, Russia) as well as at Dmitry Rogachev National Medical Research Center of Pediatric Hematology, Oncology and Immunology (Moscow, Russia), which made it possible to establish the diagnosis of LS and confirm the genetic reason for DBA.

### Immunohistochemical and Molecular Genetic Studies

**Figure 3:**
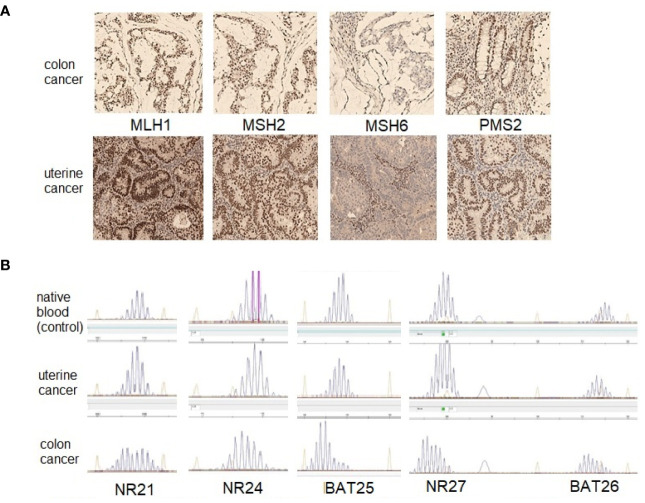
The results of investigation of the MMR status in colon and uterine tumors of patient A. **(A)**—immunohistochemical study (original magnification ×100), both tumors demonstrate lack expression of the MSH6 protein; **(B)**—fragment analysis with the panel of markers (NR21, NR24, BAT25, NR27 and BAT26).

The patient underwent an immunohistochemical study of the surgical material of uterine cancer and biopsy material of sigmoid cancer using clones ES05 (MLH1), EP51 (PMS2), FE11 (MSH2), EP49 (MSH6). The absence of expression of the MSH6 protein was found in both tumors (**Figure 3A**).

DNA from paraffin blocks for molecular genetic study was isolated using the QIAamp DNA Mini Kit according to the manufacturer’s protocol (QIAGEN, Germany). DNA from 200 µl of whole venous blood was isolated using an automatic station MagNA Pure Compact (Roche, Switzerland), according to the manufacturer’s standard protocol–DNA_Blood_100_400_V3_2. MagNA Pure Compact Software Version 1.1.2. The amount of DNA was estimated using a DeNovix QFX device (Denovix, USA) and Qubit dsDNA BR Assay Kit (ThermoFisher, USA).

Microsatellite instability (MSI) in endometrial and colon tumors was studied with five mononucleotide markers (NR21, NR24, NR27, BAT25, and BAT26) on an ABI PRISM 3500 device (eight capillaries; Applied Biosystems, USA), according to the manufacturer’s protocol. No microsatellite instability was found in the uterine tumor sample, but the colonic tumor sample revealed the MSI (**Figure 3B**).

Exome enrichment pair-end library preparation for producing Next Generation Sequencing was performed with 100 ng genomic DNA extracted from the blood. DNA sample was sheared by sonication (Covaris ME220 USA) to an average fragment size of 150 nucleotides. Hybridization with IDT xGen Exome v1 (IDT USA) oligos was an accompaniment to Truseq Exome workflow (Illumina USA). Libraries were sequenced on an Illumina NextSeq550 (Illumina USA) using 75*2 read length with dual indexing to a depth of 79,432,244 reads. Average depth of CDS regions’ coverage was 96.7 reads per bp, 87.3% of bases—with target coverage >50×. Reads were mapped using BWA (v0.7.7) (15). Variant calling and annotations were performed by SAMtools (v0.1.19) (16), GATK (1.6) (17), and dpSNP v151, GnomAD v2.1, ExAC v0.3.1, 1,000 Genomes Phase3 v5a, HGMD v2020.2 databases. Variant mining was done *via* BaseSpace Variant Interpreter (annotation engine v3.6.2.0). CNV analysis was performed with CODEX (18) and data training set (60 samples) followed by IGV (19) visualization. The obtained variants were evaluated by their occurrence in the population, effect on the amino acid sequence, location in the functional domain, scores from pathogenicity predictor programs (Sift, PolyPhen, DANN, MutationTaster, FAHMM-MKL), and other criteria according to the guidelines for interpreting human DNA sequence data obtained by mass parallel sequencing (20, 21). Assessment of the pathogenicity of CNV was also carried out according to the published criteria “Standards and guidelines for the interpretation of sequence variants: A joint consensus recommendation of the American College of Medical Genetics and Genomics and the Association for Molecular Pathology” (20) and the Clinical Genome Resource (ClinGen), with corrections for single-gene copy-number variants (21).

This allowed us to detect a clinically relevant mutation in the *MSH6* gene: NM_000179.2: c.742C>T, p.(Arg248Ter). CNV analysis showed a gross deletion (2,1 Mb) in chromosome 15 that was also visualized in the IGV genomic browser. The detected deletion included the complete sequence of the *RPS17* gene.

To find somatic mutations in the *KRAS* and *NRAS* genes (exons 2, 3, 4), we carried out PCR on a Tertsik amplifier (DNA Technology, Russia) and Sanger sequencing on ABI PRISM 3500 (eight capillaries; Applied Biosystems, USA). A somatic mutation in the *KRAS* gene was detected in a sigmoid tumor sample: NM_033360.4: c.35G>A, p.(Gly12Asp).

**Figure 4:**
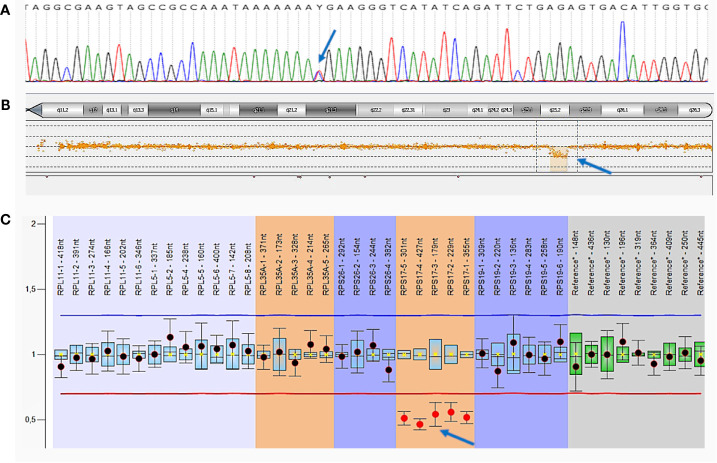
Germline mutations of patient A. **(A)**—Sanger sequencing of *MSH6* exon 4. The c.742C>T, p.(Arg248Ter) mutation is indicated by an arrow; **(B)**—comparative genomic hybridization (aCGH). Large deletion in chromosome 15 including the entire *RPS17* gene is indicated by an arrow; **(C)**—multiplex ligation-dependent probe amplification (MLPA) analysis.

The presence of a c.742C>T, p.(Arg248Ter) variant in the *MSH6* gene was confirmed by Sanger sequencing on a 3500 ABI PRISM genetic analyzer (Applied Biosystems, USA) according to the manufacturer’s protocol (**Figure 4A**).

Deletion in chromosome 15 was confirmed by comparative genomic hybridization on microchips (aCGH) with the CytoSure ™ Constitutional v3 and Constitutional v3 + LOH Arrays reagent kit 4 × 180 k format (Oxford Gene Technology, Oxfordshire) on the SureScan Microarray Scanner using Agilent Feature Extraction Software (Agilent Technologies) according to the manufacturer’s protocol. We detected a decrease in probe intensity in a 2.15 Mb area on the long arm of chromosome 15 that indicates the presence of a heterozygous deletion [arr(hg19) 15q25.2 (82,662,932–84,816,747) ×1], which includes the *RPS17* gene (**Figure 4B**).

Additionally, multiplex ligation-dependent probe amplification (MLPA) analysis was performed with a SALSA MLPA Probemix P212 DBA (MRC Holland, Netherlands) on 3500XL Genetic Analyzer (Applied Biosystems, USA), using the Coffalyser.Net software, according to the manufacturer’s protocol. The deletion of the entire *RPS17* gene was confirmed in a heterozygous state (**Figure 4C**).

### Treatment

Based on the obtained diagnostic data and taking into account the presence of synchronous colonic tumors and a confirmed diagnosis of LS, the patient underwent colectomy with end stoma formation.

The microscopic examination of the removed tissue samples revealed that the splenic flexure tumor had been presented by a moderate-differentiated adenocarcinoma with mucus-producing areas pT2N0; tumor of the sigmoid colon—by combined moderate-differentiated adenocarcinoma with foci of mucinous adenocarcinoma and signet-ring cancer pT3N0 with lymphovascular invasion; tumor of the distal third of the sigmoid—by tubulo-villous adenoma with G2 adenocarcinoma focus pT1sm2N0. No metastatic lymph nodes were found in the mesocolon; however, one of the omental lymph nodes revealed the presence of metastasis of adenocarcinoma, which was regarded as a sign of distant tumor dissemination pM1. Postoperative complications did not occur, and patient A. was discharged in satisfactory condition on day 11 after surgery.

According to oncologist`s prescriptions, the patient received two FOLFOX-6 courses as an adjuvant chemotherapy. After that, she was diagnosed with pancytopenia and as a result, the systemic chemotherapy was suspended. The patient is currently receiving symptomatic corrective treatment, and dynamic observation continues.

## Discussion

Here we present the first extremely rare observation case of a patient with two hereditary syndromes—DBA and LS, which were confirmed using several modern methods of molecular genetics: NGS, Sanger sequencing, aCGH, and MLPA. The estimated frequency of such patients worldwide, according to our estimations, is about one in 480,000,000 people, if we take into account the probability of meeting two probands with hereditary disorders (one with LS (1/750), the other with DBA (1/160,000)) and the possibility of transmitting a pathogenic mutation to a child from each of the parents [(1/750) * (1/160,000) * 1/4)]. After advanced search in available literature, we did not find any information about this combination of hereditary cancer syndromes that became the reason for publishing our data.

The proband has LS caused by a pathogenic variant in the *MSH6* gene, although mutations in patients with this syndrome in Russia are much more common in the *MLH1* and *MSH2* genes (22). The same mutation was found in the patient’s mother, who previously suffered from breast and uterine cancer. This pathogenic mutation c.742C>T, p.(Arg248Ter) in the *MSH6* gene was described by Wijnen, J. et al. in a patient with endometrial cancer (23). As for the *RPS17* gene deletion, we cannot propose its origin since the father’s biomaterial is not available because of his death at the age of 60 as a result of an accident (the mother does not have it). Deletions of the entire *RPS17* gene have been described in patients with Diamond–Blackfan anemia (24, 25). Patient A. developed endometrial cancer at the age of 36 and colorectal tumors at the age of 38 (**Figure 1B**). At the same time, her age of onset of tumors is significantly lower than the ones of her mother and grandmother, which indicates a more aggressive disease. Cases of the development of malignant neoplasms have also been described in patients not only with Lynch syndrome, but also with Diamond–Blackfan anemia (26); however, we are not able to speculate about the possible synergistic effect of mutations in *RPS17* and *MSH6* genes on the earlier age of clinical manifestations of diseases because of a single clinical experience.

It is extremely important to note that for the analysis of the patient’s uterine tumor for the presence of microsatellite instability, we used a panel that included mononucleotide markers NR21, NR24, NR27, BAT25, BAT26 (approved by ESMO 2019) (27); however, it did not detect the presence of MSI. At the same time, immunohistochemical study of the endometrial tumor unambiguously established the absence of expression of the MSH6 protein. This indicates the lack of feasibility of using the panel of markers NR21, NR24, NR27, BAT25, BAT26 to study endometrial cancer in patients with Lynch syndrome, which is caused by a mutation in the *MSH6* gene. At the same time, microsatellite instability was detected in a sample of a colon tumor both using a panel of mononucleotide markers and immunohistochemically, which indicates the equal diagnostic capabilities of these methods.

Both uterine cancer and colonic cancer samples were examined for the presence of somatic mutations in the *KRAS* and *NRAS* genes in order to determine the clonality of the development of malignant neoplasms. A mutation in the *KRAS* gene was detected in the colon tumor: NM_033360.4: c.35G>A, p.(Gly12Asp), but not in the uterine tumor. Thus, tumors most likely developed independently and from different cell clones.

## Conclusions

For the first time in the world, a clinical case of a patient with two combined hereditary diseases (Diamond–Blackfan anemia and Lynch syndrome) is presented, the estimated frequency of which is one in 480,000,000 people. The patient has well known pathogenic mutation in the *MSH6* gene: NM_000179.2: c.742C>T, p.(Arg248Ter), and a new deletion of the chromosome 15’s locus with the capture of 82,662,932–84,816,747 bp interval, including the complete sequence of the *RPS17* gene. The expediency of an immunohistochemical study, rather than a molecular genetic search for microsatellite instability in endometrial tumor samples from patients with Lynch syndrome caused by a mutation in the *MSH6* gene, has been demonstrated.

## Data Availability Statement

The datasets presented in this study can be found in online repositories. The names of the repository/repositories and accession number(s) can be found in the article/supplementary material.

## Ethics Statement

The studies involving human participants were reviewed and approved by Ethics Committee of the National Medical Radiology Research Center (Moscow, Russia). The patients/participants provided their written informed consent to participate in this study. Written informed consent was obtained from the individual(s) for the publication of any potentially identifiable images or data included in this article.

## Author Contributions

AT, DP, ER—analysis of the results and writing of the manuscript; VS, AB, LY—molecular genetic and immunohistochemical studies, VK, EF, AM, DS—patient treatment, AK, AR, NG, YS—administration and management of workflows for the project. All authors contributed to the article and approved the submitted version.

## Funding

The research was carried out within allocated funds of the State Assignment of the Russian Federation to the institutions aforementioned in this study, molecular and clinical analyses were partially funded by the “Science for Children” endowment foundation (Russian Federation).

## Conflict of Interest

The authors declare that the research was conducted in the absence of any commercial or financial relationships that could be construed as a potential conflict of interest.
